# Immunopathogenesis of *Chlamydia pneumoniae* Infections in Children

**DOI:** 10.1155/S1064744996000282

**Published:** 1996

**Authors:** Margaret R. Hammerschlag

**Affiliations:** Division of Infectious Diseases Department of Pediatrics State University of New York Health Science Center at Brooklyn New York USA


Infectious Diseases in Obstetrics and Gynecology 4:128–130 (1996)
© 1996 Wiley-Liss, Inc.

### Immunopathogenesis of *Chlamydia pneumoniae* Infections in Children

**Margaret R. Hammerschlag**

*Division of Infectious Diseases, Department of Pediatrics, State University of New York Health Science Center at Brooklyn, New York*

KEY WORDS

immunopathogenesis; *C. pneumoniae;* respiratory disease; pediatric populations

*Chlamydia pneumoniae* is emerging as a frequent cause of respiratory disease in children as well as adults. *C. pneumoniae* infection in children presents a different set of problems regarding disease presentation and diagnosis compared to adults. Several studies of the role of *C. pneumoniae* in lower respiratory tract infection in pediatric populations have found evidence of infection in 0 to over 18%.[1–8] Most of these studies have relied entirely on serology for diagnosis. Infection under the age of 5 years has been rare in Seattle and Scandinavia, whereas in a study of Filipino children under the age of 5 years presenting with lower respiratory tract infection, nearly 10% had either acute or chronic antibody to *C. pneumoniae.*[7] In Brooklyn, the proportion of lower respiratory tract infections associated with *C. pneumoniae,* as determined by culture, increased from 9% in children under the age of 5 to 19% in the 5–16-year-old age category.[8] Recent studies which have utilized culture have found a poor correlation between culture and serology, especially in children. As part of a multicenter pneumonia treatment study in children 3–12 years of age, Block et al.[1] isolated *C. pneumoniae* from 34 of 260 (13.1%) of the children enrolled. Serologic evidence of acute infection was found in 48 (18.5%), but only 8 (23%) of the culture positive children met the serologic criteria for acute infection. The serologically positive, culture negative children usually had stable high IgG titers, but no detectable IgM antibody.

Most *C. pneumoniae* infections are probably mild or asymptomatic. Initial reports emphasized mild atypical pneumonia clinically resembling that associated with *Mycoplasma pneumoniae.* In several subsequent studies, however, pneumonia associated with *C. pneumoniae* has been clinically indistinguishable from other pneumonias. Coinfection with other pathogens, especially *M. pneumoniae* and *Streptococcus pneumoniae* can be frequent. In the multicenter pneumonia treatment study 20% of the children with positive *C. pneumoniae* cultures were coinfected with *M. pneumoniae;* they could not be distinguished from those children who were infected with either organism alone.[1] The only child in this study who had pneumonococcal bacteremia also was culture positive for *C. pneumoniae. C. pneumoniae* has been associated with severe illness and even death, although the role of pre-existing chronic conditions as contributing factors in many of these patients is difficult to assess. In some cases, however, *C. pneumoniae* appears to be clearly implicated as a serious pathogen even in the absence of underlying disease. *C. pneumoniae* was isolated from the respiratory tract and the pleural fluid of a previously healthy adolescent boy with severe pneumonia complicated by respiratory failure and pleural effusions[9].

The role of host factors remains to be determined. Although *C. pneumoniae* has been detected in bronchoalveolar lavage fluid from 10% of a group of patients with AIDS and pneumonia, its clinical role in these patients is uncertain because most were coinfected with other well-recognized pathogens such as *Pneumocystis carinii* and *Mycobacterium tuberculosis.*[10] Gaydos et al.,[11] recently identified *C. pneumoniae* infection by polymerase chain reaction (PCR) in 11% of a group of immunocompromised adults with AIDS, malignancies, and other immune disorders including systemic lupus erythematosus, sarcoidosis,

Received September 15, 1996
Accepted October 1, 1996

Article and common variable immunodeficiency. We isolated *C. pneumoniae* from 6 of 31 (19%) children with sickle cell disease presenting with episodes of acute chest syndrome.[6] *C. pneumoniae* infection in these patients appeared to be associated with more severe hypoxia than infection with *M. pneumoniae.*

*C. pneumoniae* appears to act as an inflammatory trigger for asthma. There are several reports of patients with culture-documented *C. pneumoniae* infection who developed significant bronchospasm.[12,13] One patient was diagnosed as having asthmatic bronchitis and was receiving systemic and topical steroids.[13] She did not improve until her chlamydial infection was treated. Hahn et al.,[12] reported an association between serologic evidence of acute *C. pneumoniae* infection and wheezing in adults seen for lower respiratory tract illness. However, they were able to isolate the organism from only 1 of 365 patients. As part of a study in children, we isolated *C. pneumoniae* from 13 of 118 (11%) children 5–15 years of age who were initially evaluated for either new or acute exacerbations of asthma.[2] Treatment of the infection appeared to result in both clinical improvement and improvement in pulmonary function test scores in 75% of the infected children. As we observed with the children with community acquired pneumonia, only 5 of the children with confirmed infection had detectable IgG antibody to *C. pneumoniae.* One child who was noncompliant with his antibiotic therapy was culture-positive on five occasions over a 3-month period. During this time no anti-*C. pneumoniae* antibody was ever detected in his sera. We detected specific anti-*C. pneumoniae* IgE in 85.7% of the culture-positive asthmatics compared to 9% of children with *C. pneumoniae* pneumonia who were not wheezing.[14] We were also able to detect anti-*C. pneumoniae* IgE in sera of 4 patients, 9–41 years of age, with cystic fibrosis and *C. pneumoniae* respiratory infection.[15] This suggests that bronchial reactivity seen with *C. pneumoniae* infection may be IgE mediated. The potential of *C. pneumoniae* to cause prolonged, persistent infection, often for months, may produce chronic inflammation and trigger bronchospasm in susceptible individuals. Preliminary studies demonstrate that *C. pneumoniae* stimulates production of IL-6 in HEp-2 cells. IL-6 is a precourser for histamine release.

*C. pneumoniae* has been demonstrated to induce in vitro ciliostasis in ciliated bronchial epithelial cells.[16] Animal studies also suggest that steroids can reactivate *C. pneumoniae* lung infection in mice.[17] Hydrocortisone has been demonstrated to enhance the growth of *C. pneumoniae* in vitro.[18] However, hydrocortisone did not interfere with the activity of various antibiotics suggesting that steroid treatment could be continued if the patients are on appropriate antibiotic treatment. Immune-mediated phenomena, including erythema nodosum and iritis, has also been described complicating *C. pneumoniae* infection.[19,20]

As isolation of *C. pneumoniae* was difficult and initially limited, more emphasis was placed on serologic diagnosis. Grayston et al.[21] originally proposed a set of criteria for serologic diagnosis of *C. pneumoniae* infection with the MIF test that is used by many laboratories and clinicians. For acute infection the patient should have a four-fold rise in IgG titer, a single IgM titer of 1:16 or greater, or a single IgG titer of 1:512 or higher. Past or pre-existing infection is defined as an IgG titer of 1:16 or higher but less than 1:512. In initial infection, the IgM response appears about 3 weeks after the onset of illness and the IgM response appears at 6–8 weeks. In reinfection, the IgM response may be absent and the IgG occurs earlier, within 1–2 weeks. Because of the relatively long period until the development of a serologic response in primary infection, the antibody response may be missed if convalescent sera are obtained too soon, i.e., earlier than 3 weeks after the onset of illness. Use of paired sera also only affords a retrospective diagnosis, which is of little help in terms of deciding how to treat the patient. The criteria for use of a single serum sample have not been correlated with the results of culture and are based mainly on data from adults. The antibody response in acute infection may take longer than 3 months to develop. As discussed previously, acute, culture-documented infection can also occur without seroconversion, especially in children.[1,2,8] Only 28% of the culture-positive children enrolled in the multicenter pneumonia treatment study had met the serologic criteria for acute infection; most had no detectable antibody by the MIF test even after 3 months of follow-up.[1]

Subsequently, we tested sera from 46 culture positive children and 42 culture negative children with pneumonia or asthma by immunoblotting.[22] Over 80% of the culture positive, MIF negative children had antibodies to a number of *C. pneumoniae* proteins detected by immunoblot. The lack of detectable antibody by MIF in these children may be due, in part, to low reactivity to the major outer membrane protein (MOMP); only 23%–26% reacted with the MOMP. Although the MOMP has been demonstrated to be immunodominant in the immune response to *C. trachomatis* infection, and antibody to the MOMP is neutralizing, it does not appear to be so for *C. pneumoniae*.[23,24,25] Over 75% of the culture positive children reacted with a 50–52 kDa protein compared to only 43% of the culture negative children. This protein may be a heat shock protein which is probably genus-specific. However, we could not determine any specific pattern in terms of reactivity to various *C. pneumoniae* proteins in sera from culture positive and culture negative children, even with paired sera. Possible serologic cross-reactions of *C. pneumoniae* with *C. trachomatis* and other bacteria may also occur and should be investigated.

### REFERENCES

1. Block S, Hedrick J, Hammerschlag MR, Cassell GH, Craft JC: *Mycoplasma pneumoniae* and *Chlamydia pneumoniae* in pediatric community-acquired pneumonia: Comparative efficacy and safety of clarithromycin vs. erythromycin ethylsuccinate. Pediatr Infect Dis J 14:471–477, 1995.
2. Emre U, Roblin PM, Gelling M, et al.: The association of *Chlamydia pneumoniae* infection and reactive airway disease in children. Arch Pediatr Adolesc Med 148:727–732, 1994.
3. Forgie IM, O'Neill KP, Lloyd-Evans N, et al.: Etiology of acute lower respiratory tract infections in Gambian children: II. Acute lower respiratory tract infection in children ages one to nine years presenting at the hospital. Pediatr Infect Dis J 10:42–47, 1991.
4. Herrmann SB, Salih MAM, Yousi BE, et al.: Chlamydial etiology of acute lower respiratory tract infections in children in the Sudan. ACTA Paediatr 83:169–172, 1994.
5. Jantos CA, Wienpahl B, Schiefer HG, et al.: Infection with *Chlamydia pneumoniae* in infants and children with acute lower respiratory tract disease. Pediatr Infect Dis J 14:117–122, 1995.
6. Miller ST, Hammerschlag MR, Chirgwin K, et al.: The role of *Chlamydia pneumoniae* in acute chest syndrome of sickle cell disease. J Pediatr 118:30–33, 1991.
7. Saikku P, Ruutu P, Leinonen M, et al.: Acute lower-respiratory-tract infection associated with chlamydial TWAR antibody in Filipino children. J Infect Dis 158:1095–1097, 1988.
8. Chirgwin K, Roblin PM, Gelling M, Hammerschlag MR, Schachter J: Infection with *Chlamydia pneumoniae* in Brooklyn. J Infect Dis 163:757–761, 1961.
9. Augenbraun MH, Roblin PM, Mandel LJ, Hammerschlag MR, Schachter J: *Chlamydia pneumoniae* pneumonia with pleural effusion: Diagnosis by culture. Am J Med 91:437–438, 1991.
10. Augenbraun MH, Roblin PM, Chirgwin K, Landman D, Hammerschlag MR: Isolation of *Chlamydia pneumoniae* from the lungs of patients infected with the human immunodeficiency virus. J Clin Microbiol 29:401–402, 1990.
11. Gaydos CA, Fowler CL, Gill VJ, et al.: Detection of *Chlamydia pneumoniae* by polymerase-chain reaction-enzyme immunoassay in an immunocompromised population. Clin Infect Dis 17:718–723, 1993.
12. Hahn DL, Dodge RW, Golubjatnikov R: Association of *Chlamydia pneumoniae* (Strain TWAR) infection with wheezing, asthmatic bronchitis, and adult-onset asthma. JAMA 266:225–230, 1991.
13. Hammerschlag MR, Chirgwin K, Roblin PM, et al.: Persistent infection with *Chlamydia pneumoniae* following acute respiratory illness. Clin Infect Dis 14:178–182, 1992.
14. Emre U, Sokolovskaya N, Roblin PM, Schachter J, Hammerschlag MR: Detection of anti-*Chlamydia pneumoniae* IgE in children with reactive airway disease. J Infect Dis 172:265–267, 1995.
15. Emre U, Bernius M, Roblin PM, et al.: *Chlamydia pneumoniae* infection in patients with cystic fibrosis. J Infect Dis 22:819–823, 1996.
16. Shemer AY, Lieberman D: *Chlamydia pneumoniae*-induced ciliastasis inciliated bronchial epithelial cells. J Infect Dis 171:1274–1278, 1995.
17. Malinverni R, Kuo CC, Campbell LA, et al.: Reactivation of *Chlamydia pneumoniae* lung infection in mice by cortisone. J Infect Dis 172:593–595, 1995.
18. Tsumura N, Emre U, Roblin PM, Hammerschlag MR: The effect of hydrocortisone succinate on the growth of *Chlamydia pneumoniae* in vitro. J Clin Microbiol, in press, 1996.
19. Sundelof B, Gnarpe H, Gnarpe J: An unusual manifestation of *Chlamydia pneumoniae* infection: Meningitis, hepatitis, iritis and atypical erythema nodosum. Scand J Infect Dis 25:259–261, 1993.
20. Yamada S, Tsumura N, Nagai K, et al.: A child with iritis due to *Chlamydia pneumoniae* infection. J Japan Assoc Infect Dis 68:1543–1547, 1994.
21. Grayston JT, Campbell LA, Kuo CC, et al.: A new respiratory tract pathogen: *Chlamydia pneumoniae* strain TWAR. J Infect Dis 161:618–625, 1990.
22. Kutlin A, Roblin PM, Hammerschlag MR: Antibody response to *Chlamydia pneumoniae* in children with respiratory illness. In: Stary A (ed): Proceedings of the Third Meeting of the European Society for Chlamydia Research. Bologna, Societa Editice Esculapio, p 238, 1996.
23. Black CM, Johnson JE, Farshy CE, Brown TC, Berdol BB: Antigenic variation among strains of *Chlamydia pneumoniae*. J Clin Microbiol 29:1312–1316, 1991.
24. Iijima Y, Miyashita N, Kishimoto T, et al.: Characterization of *Chlamydia pneumoniae* species-specific proteins immunodominant in humans. J Clin Microbiol 32:583–588, 1994.
25. Peterson EM, Cheng X, Qu Z, de la Maza LM: Characterization of the murine antibody response to peptides representing the variable domains of the major outer membrane protein of *Chlamydia pneumoniae*. Infect Immune 64:3354–3359, 1996.
